# Cost-effectiveness of strategies to increase screening coverage for cervical cancer in Spain: the CRIVERVA study

**DOI:** 10.1186/s12889-017-4115-0

**Published:** 2017-02-14

**Authors:** Marta Trapero-Bertran, Amelia Acera Pérez, Silvia de Sanjosé, Josep Maria Manresa Domínguez, Diego Rodríguez Capriles, Ana Rodriguez Martinez, Josep Maria Bonet Simó, Norman Sanchez Sanchez, Pablo Hidalgo Valls, Mireia Díaz Sanchis

**Affiliations:** 1 Economy and Business Organisation Department, Faculty of Economics and Social Sciences, Universitat Internacional de Catalunya (UIC), Barcelona, Spain; 2 Centre for Research in Economics and Health (CRES), University Pompeu Fabra, c/Immaculada 22, 08017 Barcelona, Spain; 3 Atenció a la Salut Sexual i Reproductiva (ASSIR) SAP Cerdanyola-Ripollet, Institut Catala de la Salut, Ripollet, Barcelona, Spain; 4 Unitat de Suport a la Recerca Metropolitana Nord, IDIAP Jordi Gol. Sabadell, Barcelona, Spain; 5 Grup de Recerca GRASSIR reconegut per la Generalitat de Catalunya SGR 2014-2016, Barcelona, Spain; 6 Universitat de Barcelona, Barcelona, Spain; 7 ​Cancer Epidemiology Research Programme (CERP), ​​​Institut Català d’Oncologia-IDIBELL, L’Hospitalet de Llobregat, Barcelona, Spain; 8 Centro de Investigación Biomédica en Red (CIBERESP), Barcelona, Spain; 9 Departament de Infermeria, Universitat Autonoma de Barcelona, Bellaterra, Barcelona, Spain; 10 SAP Vallés Occidental, Institut Català de la Salut, Sabadell, Barcelona, Spain; 11 Sistemes d’Informació Sanitària, SAP Vallés Occidental, Institut Catala de la Salut, Sabadell, Barcelona, Spain

**Keywords:** Cost-effectiveness, Population screening, Cervical cancer, Increase coverage

## Abstract

**Background:**

The aim of the study is to carry out a cost-effectiveness analysis of three different interventions to promote the uptake of screening for cervical cancer in general practice in the county of Valles Occidental, Barcelona, Spain.

**Methods:**

Women aged from 30 to 70 years (*n* = 15,965) were asked to attend a general practice to be screened. They were randomly allocated to one of four groups: no intervention group (NIG); one group where women received an invitation letter to participate in the screening (IG1); one group where women received an invitation letter and informative leaflet (IG2); and one group where women received an invitation letter, an informative leaflet and a phone call reminder (IG3). Clinical effectiveness was measured as the percentage increase in screening coverage. A cost-effectiveness analysis was performed from the perspective of the public health system with a time horizon of three to five years – the duration of the randomised controlled clinical trial. In addition, a deterministic sensitivity analysis was performed. Results are presented according to different age groups.

**Results:**

The incremental cost-effectiveness ratio (ICER) for the most cost-effective intervention, IG1, compared with opportunistic screening was € 2.78 per 1% increase in the screening coverage. The age interval with the worst results in terms of efficiency was women aged < 40 years.

**Conclusions:**

In a population like Catalonia, with around 2 million women aged 30 to 70 years and assuming that 40% of these women were not attending general practice to be screened for cervical cancer, the implementation of an intervention to increase screening coverage which consists of sending a letter would cost on average less than € 490 for every 1000 women.

**Trial registration:**

ClinicalTrials.gov Identifier: NCT01373723.

**Electronic supplementary material:**

The online version of this article (doi:10.1186/s12889-017-4115-0) contains supplementary material, which is available to authorized users.

## Background

In Spain, both cervical cancer incidence and survival have remained stable over the past few years [1, 2]. The global estimate of the age-adjusted incidence rate of invasive cervical cancer was 7.8 per 100,000 woman-years in 2012, [3] which means that Spain is in the low-mid range of European countries (3.6–28.6 per 100.000 woman-years). In the Autonomous Region of Catalonia, the truncated incidence rate is 16.1 per 100,000 woman-years for those aged from 35 to 64 years, meaning the risk of developing a cervical cancer is one in 106 women who have lived to the age of 75 years old [4]. The five year net survival in Spain was 65.2 for women diagnosed during 2005–2009 and comparable with or even higher than most developed countries. Despite these relatively positive data, cervical cancer is still a public health concern because it is largely preventable and also due to the high cost of screening and treatment of cervical lesions.

Cancer cost the EU € 126 billion in 2009, with health care accounting for € 51 billion (40%) [5]. In Australia, which has lower cervical cancer incidence and higher survival than Spain, the total cost of the screening programme was estimated to be € 130.4 million (2015) and the treatment cost accounted for approximately one-third of the total (€ 109.8 million [2015]) [6].

In Spain, cytological screening for cervical cancer is largely opportunistic with some variations in the protocol according to region [7]. Eighty percent of the cases of cervical cancer in Catalonia did not undergo a previous cytology exam during the 10 years prior to diagnosis [8]. In Catalonia, the protocol, which was revised and modified by the Oncology Director Plan and the Catalan Institute of Oncology in 2006, incorporated the establishment of triennial periodicity of cytology exams in women from 25 to 65 years of age and of the HPV test in women from 40 to 65 years of age with no prior cytology exam within the previous five years or with a cytology exam carried out longer than five years previous, abnormal cytology results (no specified atypical squamous lesions), and women with post-conisation control of intraepithelial lesions. An increase in screening coverage through interventions promoting the uptake of screening should be a priority objective for health care authorities if cervical cancer cases are to be reduced and women who do not periodically have a cytology exam are to be identified.

According to a systematic review of the Cochrane collaboration, [9] evaluating interventions to stimulate the participation of women in the screening of this disease, invitations and educational interventions seem to be the most effective ways to increase participation in screening programmes. In addition, there is sufficient evidence of increasing coverage when using individualised information directed at the target population, especially with systems for call-recall (that is, SMS, email, phone calls) [10, 11]. Everett et al in 2011 encouraged providing trials to further support strategies to increase coverage [9]. This would facilitate earlier action in detecting pre-malignant lesions, helping to reduce the incidence of invasive cancer and their costs. Therefore, there is a need to evaluate strategies to increase the screening population coverage for efficiency. This will allow decision-makers to make better informed decisions on which preventive programmes to conduct in Spain. The CRICERVA study (Acera et al.: Increasing cervical cancer screening coverage: a randomised, community-based clinical trial, accepted and forthcoming) is a cluster clinical trial that assigned one of three interventions to the target population registered in the Cerdanyola SAP area in Barcelona. A total of 32,858 women residing in the study area, aged 30 to 70 years and with no record of a cervical cytology exam during the past 3.5 years, were selected. The study included four arms: three interventions (a personalised invitation letter, an additional informative leaflet, and an additional personalised phone call) and a control group (based on spontaneous demand).

The aim of this study is to perform a cost-effectiveness analysis alongside the CRICERVA clinical trial, (Acera et al.: Increasing cervical cancer screening coverage: a randomised, community-based clinical trial, accepted and forthcoming) of three different active interventions to promote the uptake of screening for cervical cancer in general practice. An orientative protocol of this economic evaluation was first published in 2011 [12].

## Methods

### CRICERVA project

The CRICERVA Study was a community-based cluster clinical trial with four arms assigned into groups and performed in a predefined geographical area as defined by the Primary Health Care Service (SAP) Cerdanyola, in the metropolitan belt of Barcelona, Spain, and was subdivided into five areas, four of which were included in this study. SAP Cerdanyola covers a population of 120,293 individuals over the age of 14 years. The female population aged between 30–70 ascribed to the study areas were as follows, Study Area 1: *N* = 8968; Study Area 2: *N* = 8169; Study Area 3: *N* = 11,027; and Study Area 4: *N* = 4694. For the study’s purposes, the eligibility criteria included women from 30 to 70 years of age whose general practitioner was ascribed to the SAP Cerdanyola area, who were residents of the area for more than six months, and who had no record in the medical registry of screening for cervical cancer in the prior 3.5 years. This resulted in the identification of 15,965 out of 32,858 (48.58%) women. The selected women were clustered randomly and contacted according to their allocated arm. When personal contact was established, they were asked to answer interview questions. Additional file 1 presents the questionnaire developed for this study. The interview allowed us to identify those women appropriate for screening and invite them for testing.

The sample size was calculated based on the detection of a difference in effectiveness compared with the NIG. It was calculated by multiplying the size of a simple randomised design by the design effect or factor of inflation. For the simple randomised design, accepting an alpha risk of 0.05 and a beta risk of 0.20 in a bilateral contrast, 59 subjects were required in the first group and 59 in the second group to detect a difference of greater than or equal to 28.4% in the screening coverage of 41.6% in the NIG. The lost follow-up rate was estimated at 20%. The calculation of the sample was performed using the Granmo 5.2 computer programme for Windows. Considering an intraclass correlation coefficient of 0.05 and a mean number of 3500 women from 30 to 70 years of age with incorrect screening by Basic Health Care Area, the design effect was 176 and, thus, 20,768 women with incorrect screenings were required. Women eligible for screening were verbally informed about the screening procedures and the significance of the results. Women were excluded if they had a hysterectomy, a current history of cervical intraepithelial lesions, carcinoma in situ and cervical uterine cancer, and a diagnosis of HIV or immunosuppression. All members of the targeted population were invited to participate. AP Cerdanyola was divided into five Basic Health Care Centres (BHCC), four of which were included in the study.

The cluster unit was each of the four BHCCs. Each of the four participating BHCCs were randomly assigned to one study arm. The follow-up period of this trial finished when the diagnosis of each screening visit was completed. After completing the recruitment of the intervention groups, we characterised the women in the NIG in terms of screening practices and, if appropriate, invited them to be screened.

The interventions evaluated were 1) a personalised invitation letter to participate in the screening signed by the patient’s primary care physician and professionals of the corresponding Public Health Center (IG1); 2) the same letter of invitation sent in IG1 as well as an informative leaflet on the prevailing screening of cervical cancer (IG2); and 3) the same intervention as the one performed in IG2, complemented with a phone call three days prior to the appointment indicated in the letter of invitation as a reminder of the visit (IG3). These three interventions were compared to the NIG in which participants could avail of current opportunistic screening. There was one common action in the three different interventions, which was scientifically validated as effective, and consisted of a personalised invitation letter sent by the primary health care professionals including a fixed appointment with the GP to get a cytology test. Two other different interventions (informative leaflet and reminder call) were also included to evaluate approaches for which there are few studies assessing the effectiveness of attendance of screening programmes. Women were distributed as follows: 4197 patients to IG1; 3601 to IG2; 6088 to IG3; and 2079 to the NIG. Sociodemographic characteristics of the population are shown in Table 3. From these, 1377 (47%) women in IG1, 1258 (48%) in IG2, and 1628 (40%) in IG3 did not meet the appointments. In addition, 1248 women in IG1, 976 in IG2, and 2064 in IG3 were excluded because of adequate screening in the private system, a hysterectomy, a cervical disease, a change of address, or death. These numbers add up to 1578 screening visits in IG1, 1367 visits in IG2, and 2396 visits in IG3. Hence, the average total number of patients who responded to all the interventions was approximately 56%. The highest response rate was observed in the IG2 group (58.3%), followed by IG1 (55.9%), and IG3 (53.7%). The youngest (younger than 40 years) and the elderly (70 years or older) were the groups least responsive to any intervention. Table 1 shows the target population; women invited to participate in this study because the last screening happened three and a half years prior; women who were contacted and were willing to attend the GP visit; and the number of women who finally attended the GP visit.

**Table 1:** Population included in the CRICERVA project

Population	IG1 (letter)	IG2 (letter + leaflet)	IG3 (letter + leaflet + phone call)
Target population			
< 40	3251	2847	3799
40-49	2444	2146	2812
50-59	1784	1900	2406
≥ 60	1489	1276	2010
Total	8968	8169	11027
Poorly screened population^a^			
< 40	1113	948	1449
40-49	1224	974	1750
50-59	798	754	1260
≥ 60	1062	925	1629
Total	4197	3601	6088
Answer to the intervention^b^			
< 40	879	862	1079
40-49	861	683	1050
50-59	611	589	932
≥ 60	604	491	963
Total	2955	2625	4024
Women screened by the intervention^c^			
< 40	449	392	576
40-49	512	381	665
50-59	314	318	584
≥ 60	303	276	571
Total	1578	1367	2396

^a^Invited to participate because last screening was more than three and a half years ago

^b^Those women who are contacted through any of the interventions and are willing to attend the GP visit

^c^Number of women who finally attend th GP visit

The Ethical Committee of the Institute of Research in Primary Care (IDIAP Jordi Gol) in Catalonia, Spain, approved this study, as well as the CRICERVA study (Acera et al.: Increasing cervical cancer screening coverage: a randomised, community-based clinical trial, accepted and forthcoming).

### Health outcome and cost data

Effectiveness data were provided from the CRIVERVA project (Acera et al.: Increasing cervical cancer screening coverage: a randomised, community-based clinical trial, accepted and forthcoming). The outcome measure was the increase percentage in the screening coverage over 42 months. The acceptance rate was highest among the IG3 group (23%), followed by IG1 (18.6%), while IG2 had the lowest average success rate (17.4%).

The analysis was performed from the Public Health System perspective and, therefore, only direct health care costs were included. All available management costs per unit were adapted from Diaz et al., [13] whereas strict costs from interventions were calculated from the Reproductive and Sexual Health Primary Care Unit (ASSIR) [14] (Table 2). Management costs included 15 min for a nurse or midwife visit, a cytology kit for taking the smear, and an HPV test. Inflation rates were applied to management costs in 2014 [15]. These three costs were considered in the three interventions and also for the NIG, because all women – opportunistically or not – coming to the Basic Health Care Area (BHCA) were incurring these costs. However, the costs for each of the interventions were different. The IG1 included the costs of a letter, postage, and two minutes of an officer’s time to prepare the letter for posting. The IG2 included the costs of IG1, plus the cost of a leaflet and just a few more seconds of the officer’s time to prepare this mail-out. Finally, the IG3 included not only the costs of IG2, but the cost of a reminder call lasting one to five minutes and the extra officer time spent carrying this out. Costs are expressed in €, 2014.

**Table 2:** Management costs

Interventions	Costs(€ 2014)
NIG	Includes one visit with 15 min of a nurse/midwife (35.64€), one citology (21.78€) and one HPV test (28.71€) (Total: 86.13€)
IG1	Cost of the no intervention plus a letter and its posting (0.16€) and the office time (0.33€) (Total: 86.62€)
IG2	Cost of the no intervention plus a letter and its posting (0.16€) plus a leaflet and its posting (1€),and the officer time (0.33€ + 0.35€) (Total: 87.97€)
IG3	Cost of the no intervention plus a letter and its posting (0.16€) plus a leaflet and its posting (1€),a reminding call (0.30€) and the officer time (0.33€ + 0.35€ + 0.83€) (Total: 89.10€)

*Source:* References [12] and (Acera et al.: Increasing cervical cancer screening coverage: a randomised, community-based clinical trial, accepted and forthcoming)

### Analysis

The time horizon of the analysis was 3.5 years, the duration of the randomised controlled clinical trial. Costs and effects were not discounted because the results are reported over the trial period. A cost-effectiveness analysis of the different interventions was performed using incremental cost-effectiveness ratios (ICERs) [16].

ICERs were calculated as the additional benefit to be gained in € per effectiveness unit (1% coverage) from one alternative compared to another.

$$ \frac{\mathrm{Difference}\ \mathrm{in}\ \mathrm{Costs}\ \mathrm{Between}\ \mathrm{Two}\ \mathrm{Interventions}}{\begin{array}{l}\mathrm{Difference}\ \mathrm{in}\ \mathrm{the}\ \%\ \mathrm{of}\ \mathrm{Screening}\ \mathrm{Coverage}\hfill \\ {}\kern4em \mathrm{Between}\ \mathrm{Two}\ \mathrm{Interventions}\hfill \end{array}} $$

All results were presented according to different age groups (<40; 40–49; 50–59; ≥60). In order to measure the uncertainty of results, a deterministic univariate sensitivity analysis was performed to examine the effect of the uncertainty on the effectiveness parameter.

## Results

Table 3 describes the sociodemographic and behavioural characteristics of women interviewed in each intervention group. Table 4 presents the cost-effectiveness analysis of the intervention groups. The ICERs’ competing choices approach shows that, including women of all ages, IG2 is strongly dominated because it is more expensive and less effective than IG1. IG1 costs € 2.78 per 1% increase in coverage compared to an opportunistic screening and IG3 costs € 13.73 per 1% increase in coverage more than an opportunistic screening, making IG1 more cost-effective. In the comparisons with the next best alternative, IG3 costs € 60.73 per 1% increase in coverage more than IG1. Therefore, for women of all ages, IG1 is the most cost-effective alternative. Results differ in scale across age groups, but not conceptually and IG2 is always strongly dominated by IG1 (see Table 5). ICERs for IG1, compared with opportunistic screening or the next best alternative are lower than € 4 per 1% increase in coverage for all age groups. IG2 costs € 103.85 per 1% increase in coverage more than IG1 for women ≥60 years; for the rest of the age groups, IG2 compared to IG1 is either a dominated or more expensive alternative. The age group obtaining worst results in terms of efficiency was women aged <40 years, although ICERs are still quite economically sensible (€ 3.55 per 1% increase in coverage for IG1 and € 177.86 per 1% increase in coverage for IG3). Therefore, consistently sending a letter seems to be the most cost-effective intervention for women of all ages.

**Table 3:** Sociodemographic and behavioural characteristics of women interviewed

Characteristic	No Intervention Group (NIG)	Intervention groups			Total	*P*
		Letter (IG1)	Letter + leaflet (IG2)	Letter + leaflet + phone call (IG3)		
Interviewed	857	807	848	1011	3523	
Age, mean (SD)	50.8 (12.7)	49.5 (12.1)	50.0 (12.4)	51.1 (12.0)	50.4 (12.3)	0.018
Spanish nationality	827 (96.5%)	744 (92.2%)	768 (90.7%)	900 (89.1%)	3239 (92.0)	<0.001
Educational level						
None	43 (5.1%)	82 (11.9%)	64 (9.5%)	71 (8.0%)	260 (8.4%)	<0.001
Primary	504 (30.1%)	380 (27.5%)	377(28.0%)	423 (23.8%)	1684 (27.2%)	
High School/ University	291(17.3%)	229(16.5%)	231(17.2%)	395(22.2%)	1146(18.5%)	
Marital status-married	594 (70.3%)	518 (74.6%)	513 (76.5%)	666 (74.7)	2291 (73.9%)	0.037
Number of children						
0	93 (11.0%)	97 (14.0%)	74 (11.0%)	114 (12.8%)	379 (12.2%)	0.002
1-2	443 (52.5%)	404 (58.4%)	394 (58.5%)	524 (58.9%)	1765 (57.0%)	
> 2	308 (36.5%)	191 (27.6%)	205 (30.5%)	251 (28.2%)	955 (30.8%)	
Lag time since last Pap screening						
1-3 years	417 (48.7%)	348 (43.8%)	369 (44.6%)	421 (42.1%)	1555 (44.7%)	0.002
4-6 years	322 (37.6%)	282 (35.5%)	294 (35.6%)	391 (39.1%)	1289 (37.1%)	
never	117 (13.7%)	164 (20.7%)	164 (19.8%)	189 (18.9%)	634 (18.2%)	
Reasons for non-attendance to screening for women with no previous Pap						
Fear and dislike	23 (19.8%)	65 (41.1%)	68 (42.2%)	73 (40.3%)	229 (37.2%)	<0.001
Uninformed	91 (78.4%)	84 (53.2%)	80 (49.7%)	98 (54.1%)	353 (57.3%)	
Other	2 (1.7%)	9 (5.7%)	13 (8.1%)	10 (5.5%)	34 (5.5%)	

The questionnaires completed for the intervention groups were carried out during routine medical visits. For the non-intervention group the questionnaires were completed at the end of the study by appropriately trained personnel during a telephone call

*Source:* (Acera et al.: Increasing cervical cancer screening coverage: a randomised, community-based clinical trial, accepted and forthcoming)

**Table 4:** Cost-effectiveness analysis results over the CRICERVA study for all ages

Group	Cost	Incremental coverage (%)	ICER(1)	ICER(2)
No intervention (NIG)	86.13€			
IG1 (letter)	86.62€	17.6%	2.78	2.78 (IG1 vs NIG)
IG2 (letter + leaflet)	87.97€	16.7%	11.02	Dominated (IG2 vs IG1)
IG3 (letter + leaflet + phone call)	89.11€	21.7%	13.73	60.73 (IG3 vs IG1)

(1) Incremental cost-effectiveness ratio of each intervention group compared with the no intervention (opportunistic screening) group expressed as € per 1% coverage

(2) Incremental cost-effectiveness ratio of one intervention compared with the next least expensive strategy expressed as € per 1% coverage

**Table 5:** Cost-effectiveness analysis results over the CRICERVA study by age group

Age	Incremental coverage (%)	ICER(1)	ICER(2)
Women < 40			
No intervention (NIG)			
IG1 (letter)	13.8%	3.55	3.55 (IG1 vs NIG)
IG2 (letter + leaflet)	13.8%	13.33	more expensive (IG2 vs IG1)
IG3 (letter + leaflet + phone call)	15.2%	19.60	177.86 (IG3 vs IG1)
Women 40-49			
No intervention (NIG)			
IG1 (letter)	20.9%	2.34	2.34 (IG1 vs NIG)
IG2 (letter + leaflet)	17.8%	10.34	Dominated (IG2 vs IG1)
IG3 (letter + leaflet + phone call)	23.6%	12.63	92.22 (IG3 vs IG1)
Women 50-59			
No intervention (NIG)			
IG1 (letter)	17.6%	2.78	2.78 (IG1 vs NIG)
IG2 (letter + leaflet)	16.7%	11.02	Dominated (IG2 vs IG1)
IG3 (letter + leaflet + phone call)	24.3%	12.26	37.16 (IG3 vs IG1)
Women ≥ 60			
No intervention (NIG)			
IG1 (letter)	20.3%	2.41	2.41 (IG1 vs NIG)
IG2 (letter + leaflet)	21.6%	8.52	103.85 (IG2 vs IG1)
IG3 (letter + leaflet + phone call)	28.4%	10.49	16.76 (IG3 vs IG1)

(1) Incremental cost-effectiveness ratio of each intervention group compared with the no intervention (opportunistic screening) group expressed as € per 1% coverage

(2) Incremental cost-effectiveness ratio of one intervention compared with the next least expensive strategy expressed as € per 1% coverage

### Sensitivity analysis

When the increase of coverage is reduced by 50%, results remain the same in terms of efficiency ranking, with the option of sending a letter (IG1) being the most cost-effective intervention compared with doing nothing. Even if the final coverage was decreased by 75% of the results experienced in the CRICERVA study, cost-effectiveness results would remain, showing the robustness of this analysis and the low values obtained for ICERs of each intervention compared with doing nothing.

## Discussion

This economic evaluation assessed whether the increase in participation rates of screening for cervical cancer compensates for the costs incurred from different interventions. Observing our results, if a universal strategy is applied for all age groups, the preventive intervention of sending a letter for an appointment is the most efficient with a cost of around € 3 per 1% coverage, followed by sending a letter with a leaflet and a reminder call with a cost of € 61 per 1% coverage. The intervention of sending a letter with a leaflet (IG2) is more expensive and less effective than only sending a letter (IG1). Results by age are consistent; the intervention of sending a letter costs less than € 4 per 1% coverage and sending a letter with a leaflet and a reminder call costs between € 2 and € 178, depending on the age; the older the women, the more cost-effective this intervention.

The cost-effectiveness of interventions to promote cervical cancer have already been assessed in another study [17]. Although not all the interventions were the same as the ones analysed in this paper, the letter was common to all of them and the comparator was the opportunistic screening. In that paper, the most cost-effective intervention was to remind a doctor to offer a smear during a consultation; however, the authors were operating in a relatively disadvantaged area and populations are not comparable. However, another paper reinforces the results we obtained in this study [18]. The authors state that telephone contact with women who have abstained from cervical cancer screening for a long time increases participation and leads to a significant increase in detection of atypical smears. Other authors also support the idea that contacting women through a postal reminder is as effective as, and less expensive than, a telephone call [19]. In our study, there was no intervention involving an email, but IG3 comprised a telephone call and was the least cost-effective intervention compared to opportunistic screening.

According to some authors, there are large variations in cervical cancer screening policies, coverage, and quality of screening across Europe [20]. As assessed by other studies, the recommendations of the Council of the European Union (EU) on organised population-based screening for cervical cancer have not yet been fulfilled [21]. The European cervical cancer screening guidelines were prepared for all European countries (not just for EU members), but many of them failed in implementation [22]. Spain has opportunistic screening implemented by regions and the age range is established between 30 and 65 years [20]. Decisions on the target age group and frequency of screening are usually made at the national level; however, continued unavailability of population-based, systematically organised screening programmes to women who may benefit from screening remains the major obstacle in the control of cervical cancer in Europe. Some authors claim that the evaluation of screening activity related to cervical cancer using cohort studies designs among screening populations are proceeding in some countries, but results are not available yet [23]. Others recently stated that a shift from opportunistic to organised screening is imperative to optimise the cost and impact of screening, but no evidence on cost-effectiveness has been published for this type of study [24]. This paper tries to bridge these gaps by providing information on efficiency of different interventions in order to start building a nationally organised screening programme. However, the available evidence supports the hypothesis that while organised population screening programmes are successful in increasing overall participation rates, they may not *per se* substantially reduce social inequalities [25].

With regard to the factors influencing participation in screening, some authors have suggested the following: the absence of population programmes; low sensitisation with respect to preventive attitudes in cohorts of elderly women; and health care overload in primary care centres [10, 26].

This economic evaluation just covers diagnosis on the illness pathway; however, this will influence the cost-effectiveness of the whole cervical cancer pathway. Therefore, there is a need to build a model for the natural history of cervical cancer for Spain, such as the one built for Germany [27] and study the cost-effectiveness of the whole pathway, accounting for organised cervical cancer screening programmes.

In a population like Catalonia with around two million women aged 30–70 years and assuming that 40% (800,000) of these women have not been screened for the last 3 years, the implementation of an intervention to increase screening coverage in 1% implies that the government would need to pay € 2.78 for a 1% of increase in coverage. In this study, the NIG (*n* = 428 women, spontaneous demand) cost the Catalonian government €36,864; the women attending the screening because of the letter (IG1, *n* = 1578) cost € 136,683 (which increased the coverage of the screened population by 17.6%); the women attending the screening because of the letter and the leaflet (IG2, *n* = 1367) cost € 120,255 (which increased the coverage of the screened population by only 16.7%); and the women attending the screening because of the letter, leaflet, and phone call (IG3, *n* = 2396) cost € 213,484 (which increased the coverage of the screened population by 21.7%). However, if all women were contacted using IG1, the most cost-effective strategy, the screening of the 5669 women would have cost € 491,049; therefore, the Catalonian government would have saved € 16,237. Obviously, the higher the number of women screened, the higher the saving. Thus, to test 5669 women costs € 507,286 in total.

## Conclusion

The ICER for the most cost-effective intervention, IG1, compared with opportunistic screening was € 2.78 per 1% increase in the screening coverage; IG2 and IG3 have more efficiency for the elderly group (≥60). Sending a letter would cost on average around € 490 for every 1000 women. The age interval with the worst results in terms of efficiency was for women aged <40 years. This analysis encourages including this intervention in the national policy on screening to prevent cervical cancer, because this would complement the opportunistic system; until then, screening cannot be said to be organised at a national level.

## Additional file

Additional file 1: Cricerva Study Questionnaire. Description of data: Interview guide developed during the study. (PDF 36 kb)

## Abbreviations

ASSIR: Reproductive and sexual health primary care unit
BHCA: Basic health care area
HPV: Human papillomavirus
ICER: Incremental Cost-Effectiveness Ratio
IDIAP: Primary Care Research University Institute
IG1: Intervention Group 1 which included participants to whom a personalised invitation letter to participate in the screening signed by the patient’s primary care physician and professionals of the corresponding public health centre was sent
IG2: Intervention Group 2 which included participants to whom the same letter of invitation in IG1 was sent, as well as an informative leaflet on the prevailing screening of cervical cancer
IG3: Intervention Group 3 which included participants who received the same interventions as those performed in IG2, complemented by a phone call three days prior to the appointment indicated in the letter of invitation as a reminder of the visit
NIG: No intervention group; participants in this group had access to current opportunistic screening
SAP: Primary care service
