# Supplementary material for: Cost-effectiveness of strategies to increase screening coverage for cervical cancer in Spain: the CRIVERVA study
Source: BMC Public Health. 2017 Feb 14;17:194. doi: 10.1186/s12889-017-4115-0 (PMC5309977; doi:10.1186/s12889-017-4115-0)
Supplement: Additional file 1: — Cricerva Study Questionnaire. Description of data: Interview guide developed during the study. (PDF 36 kb) [file 12889_2017_4115_MOESM1_ESM.pdf]

CRICERVA STUDY QUESTIONNAIRE

1. Did not attend the first visit
2. Nationality
3. Pathological antecedents
   - ☐ None
   - ☐ Hysterectomy
   - ☐ Uterine cancer
   - ☐ Cervical cancer
   - ☐ Other
   - ☐ Don't know /don't answer
4. Have you ever had a Pap smear? If yes, how often?
   - ☐ Never
   - ☐ Every year
   - ☐ Every 2 years
   - ☐ Every 3 years
   - ☐ Every 4 years
   - ☐ Every 5 years or more
   - ☐ I don't know / I do not wish to answer
5. If you had your last cytology test more than three years ago, what was the reason?
   - ☐ I did not like it
   - ☐ I'm afraid
   - ☐ I didn't think it was not important
   - ☐ I did not know that it was necessary
6. Where was the cytology performed?
   - ☐ Public health system
   - ☐ Private sector
   - ☐ Mixed (public and private)
   - ☐ I don't know / I do not wish to answer
7. Have you ever had a previous HPV test?
   - ☐ Yes
   - ☐ No
   - ☐ I don't know / I do not wish to answer
8. Are you in a relationship?
   - ☐ Yes
   - ☐ No
   - ☐ I don't know / I do not wish to answer
9. Number of children _______.
10. Number of caregivers _______.
11. What is your highest level of education?
    - ☐ I have no formal education
    - ☐ Primary, incomplete
    - ☐ Primary
    - ☐ Secondary
    - ☐ Tertiary education (university)
    - ☐ I don't know / I do not wish to answer
12. What is your current employment situation?
    - ☐ Employed
    - ☐ Domestic worker
    - ☐ Student
    - ☐ On sick leave
    - ☐ Provisional disability
    - ☐ Retired
    - ☐ I don't know / I do not wish to answer
13. Monthly income of the woman
    - ☐ 0€ to 600€
    - ☐ 601€ to 1000€
    - ☐ 1001€ to 2000€
    - ☐ More than 2000€
    - ☐ I don't know / I do not wish to answer
14. Monthly income of the family
    - ☐ 0€ to 600€
    - ☐ 601€ to 1000€
    - ☐ 1001€ to 2000€
    - ☐ More than 2000€
    - ☐ I don't know / I do not wish to answer
